# Stress and sex in malaria parasites: Why does commitment vary?

**DOI:** 10.1093/emph/eot011

**Published:** 2013-06-04

**Authors:** Lucy M. Carter, Björn F.C. Kafsack, Manuel Llinás, Nicole Mideo, Laura C. Pollitt, Sarah E. Reece

**Affiliations:** ^1^Institute of Evolutionary Biology, School of Biological Sciences, Ashworth Laboratories, University of Edinburgh, Edinburgh, UK; ^2^Lewis-Sigler Institute for Integrative Genomics, Princeton University, Princeton, NJ, USA; ^3^Department of Molecular Biology, 246 Carl Icahn Lab, Washington Road, Princeton University, Princeton, NJ, USA; ^4^Center for Infectious Disease Dynamics, Departments of Biology and Entomology, Pennsylvania State University, Millennium Science Complex, University Park, PA, USA and ^5^Centre for Immunity, Infection & Evolution. Institutes of Evolution, Immunology and Infection Research, School of Biological Sciences, Ashworth Laboratories, University of Edinburgh, Edinburgh, UK

**Keywords:** Plasmodium, transmission, commitment, stress, phenotypic plasticity, gametocyte

## Abstract

For vector-borne parasites such as malaria, how within- and between-host processes interact to shape transmission is poorly understood. In the host, malaria parasites replicate asexually but for transmission to occur, specialized sexual stages (gametocytes) must be produced. Despite the central role that gametocytes play in disease transmission, explanations of why parasites adjust gametocyte production in response to in-host factors remain controversial. We propose that evolutionary theory developed to explain variation in reproductive effort in multicellular organisms, provides a framework to understand gametocyte investment strategies. We examine why parasites adjust investment in gametocytes according to the impact of changing conditions on their in-host survival. We then outline experiments required to determine whether plasticity in gametocyte investment enables parasites to maintain fitness in a variable environment. Gametocytes are a target for anti-malarial transmission-blocking interventions so understanding plasticity in investment is central to maximizing the success of control measures in the face of parasite evolution.

## INTRODUCTION

*Plasmodium spp* (malaria parasites) and other Apicomplexans are some of the most serious pathogens of humans, livestock and wildlife [1]. Cycles of asexual replication inside host red blood cells (RBCs), lasting from 24 to 72 hours [2], enable parasites to establish and maintain infections. To transmit to new hosts, every cell cycle a proportion of parasites develop into specialized sexual stages called gametocytes, which do not replicate in the host, but are infectious to the mosquito vector (unlike asexual stages). When taken up by the vector, male and female gametocytes differentiate into gametes and mate. The resulting offspring infect the vector and eventually produce stages infective to new hosts [3].

It is well known that the production of gametocytes varies during infections and across hosts [4–7]. However, the factors that induce commitment to produce gametocytes, and why parasites respond to these factors, are long-standing questions [8–11]*.* This information is central to understanding severity and transmission of disease, for predicting how disease control strategies will affect infectiousness [12–15], and may also reveal novel ways to target parasites.

Here, we propose that malaria parasites strategically adjust investment into gametocytes (hereafter, the conversion rate) in response to the changeable conditions experienced during infections and that plasticity in the conversion rate enables parasites to optimize their survival and transmission during infections. Our conceptual model stems from the integration of diverse experimental data into an ecological and evolutionary framework, thereby making the predictions of our model and its underlying assumptions explicit and testable. While we focus on malaria parasites, the concepts and approach we outline can be applied more broadly to species for which in-host replication and between-host transmission are achieved by different specialized stages.

## CONVERSION RATE: EVOLUTIONARY CONTEXT

Parasites experience rapid and extensive variation in their in-host environment (e.g. in resource availability, competition with other genotypes and species, immune responses, and drug treatment) throughout their infections and while occupying different hosts and vectors. There is mounting evidence that traits underpinning in-host replication and between-host transmission (spanning from immune evasion traits [16, 17] to investment in transmissible forms [4, 18, 19]) are adjusted by parasites during infections. This flexibility in traits is called ‘phenotypic plasticity’ defined as the ability of a genotype to produce different phenotypes in response to environmental change [20, 21]. Phenotypic plasticity is an important solution to the challenges of life in a changing environment because it enables organisms to maintain fitness by altering their phenotype, through mechanisms such as differential gene expression, to match their circumstances [22].

Every cell cycle malaria parasites face a resource allocation trade-off between how much to invest in asexual stages that are required for in-host survival and in sexual stages that are essential for between-host transmission [23, 24]. This is analogous to the trade-off between survival and reproduction faced by all sexually reproducing organisms [25, 26]. Because reproduction is costly, phenotypic plasticity in the conversion rate influences two key fitness components: in-host survival and between-host transmission [24]. High conversion early in infections increases the potential for transmission, but this strategy risks insufficient investment in asexual stages to maintain the infection within the host, resulting in a short duration for transmission. Conversely, excessive investment in asexual parasite replication reduces the rate of transmission at any given time, but this may be compensated for by longer infection durations and continued opportunities for transmission [24, 27].

**Figure 1.**
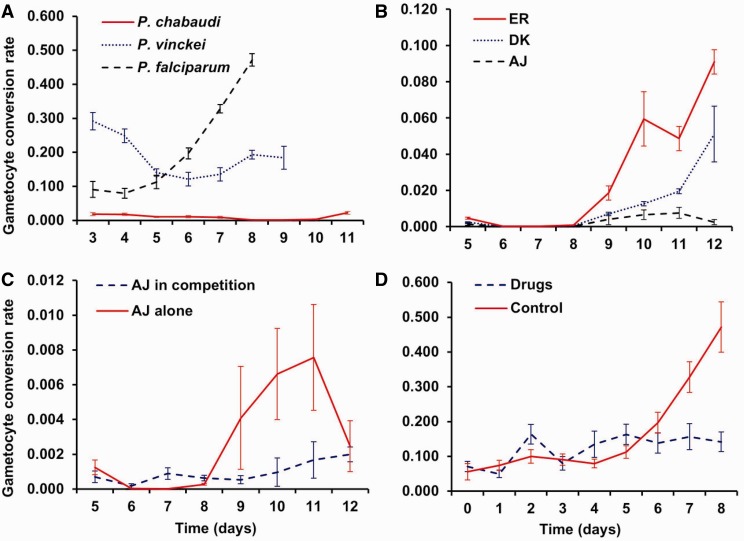
*Plasmodium* conversion rates are variable. The conversion rate (±SEM) represents the proportion of a given cohort of asexual parasites that differentiate into sexual stage gametocytes. Variation in conversion is observed across species and during infections/culture (**A**). *Note*: conversion is calculated differently for rodent malaria parasites (*P. chabaudi, P. yoelli, P. vinckei and P. berghei, in vivo*) and for *P. falciparum* (*in vitro*) (see Box 1). Different conspecific genotypes of *P. chabaudi*, in the same experiment, exhibit different patterns for conversion during infections (**B**). *Plasmodium chabaudi* reduces conversion when experimentally exposed to in-host competition (**C**). The conversion rates of genotype AJ are illustrated; during a single genotype infection (alone), and the mean conversion when in competition with either genotypes ER, AS, or both together (in competition). The reduction in conversion observed when drug sensitive *P. falciparum* isolates are exposed *in vitro* to antimalarial drugs or control conditions (**D**) [7, 37, 39]

The number of gametocytes produced during infections is generally low [9] and it has been suggested that high densities of asexual stages are needed to shield gametocytes from transmission blocking immune responses [28]. However, this hypothesis does not explain why conversion rates vary during infections, between conspecific genotypes, and across species [7, 37, 39] (Fig. 1). The conversion rate is defined as the proportion of asexual stage parasites that commit to producing gametocytes in subsequent cell cycles (Box 1), and is called ‘reproductive effort’ in evolutionary biology. Therefore the conversion rate is not synonymous with the density or prevalence of gametocytes; variation in gametocyte densities can be generated by the same level of investment from different numbers of asexual stages [6].

**Box 1: Calculating conversion rates**

Current protocols for *in vitro* studies of *P. falciparum* calculate the conversion rate on day *t* as the number of stage II gametocytes observed in 10 000 RBCs on day *t* + 3 (the earliest time point when *P. falciparum* gametocytes are distinguishable from asexual blood stages) divided by the number of ring-stage asexual parasites observed in 10 000 RBCs on day *t* [83].

For *P. chabaudi*, conversion is calculated from *in vivo* measurements according to [6]. The description of the biological process underlying the model in [6] overcomes challenges posed by hard-to-quantify parameters (i.e. parasite death rates in the bloodstream and schizont burst sizes) and takes into account the maturation times of gametocytes and asexual blood stages (48 and 24 hours respectively, for rodent parasites). Although the mathematical formulation assumes gametocytes are counted 24 hours into development, current molecular assays count gametocytes of an unknown age (but are likely to be between 24 and 48 hours old). Ideally we need to know the schedule of development and the precise point at which gametocytes are assayed, since these will determine the exact form of the conversion rate equation. For example, if markers in mature (48 h old) gametocytes are used, then conversion rate, *ε,* should be quantified as:

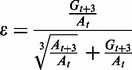

where *A_i_* and *G_i_* are asexual and gametocyte densities on day *i*.

While these tools are easy to implement, the assumptions underpinning them are key to making accurate estimates of conversion rates. These assumptions, and their caveats, include:

1. The probability of asexual parasites producing gametocytes is constant over the period between gametocyte production and detection. Given the expectation of plasticity in conversion, whereby a different proportion of asexual parasites can commit for every cell cycle, this assumption may often be hard to fulfil.
2. Both *in vivo* and *in vitro* approaches assume that the death rate of asexual parasites and gametocytes is equal. Whilst *in vitro* culture conditions do not have the problem of sequestration (disappearance from the circulation) or immune factors that could exacerbate differential mortality rates between lifecycle stages [9], for *in vivo* assays these factors could confound conversion estimates [40]. Furthermore, conversion rates can be overestimated if the death rate for asexual parasites is higher than for gametocytes (which could well be the case during drug treatment [39], or underestimated if early stage gametocytes are mistakenly identified as asexual stages. It is possible to develop mathematical models and formulate predictions for how different survival rates need to be if they are the sole driver of observed patterns in conversion rates. For example for the *in vivo P. chabaudi* data in [7], we find that the difference in survival rates between asexual parasites and gametocytes must vary over the course of infections (e.g. immunity sometimes focuses efficiently on killing gametocytes while at other times survival rates across parasite stages are equal) and must vary considerably in different kinds of infection (N. Mideo, unpublished results). In particular, to explain the difference in patterns of conversion observed in Fig. 1C, survival rates of gametocytes (relative to asexual parasites) in mixed infections must be several orders of magnitude lower than in single infections. As yet, there is no known mechanism that could underlie such drastically different patterns of survival between parasite stages, during and across infections. Therefore, we propose that differential survival is unlikely to be the sole cause of variation in patterns of conversion rates. However, developing a better understanding of immune responses and subsequent parasite death rates remains an important goal.

In the literature, there are considerable discrepancies in how conversion rates for *P. falciparum* have been examined, with some studies measuring the gametocyte density in circulation and others presenting gametocyte prevalence (reviewed in [12]). This is, in part, due to the difficulties in calculating conversion rates for natural *P. falciparum* infections since repeated samples—at specific time points—are required to assay the number of asexual parasites in a cohort and the number of gametocytes they produce.

Basing inference simply on gametocyte density can be problematic: for example, observations of elevated gametocyte densities post drug treatment could be due to the release of sequestered gametocytes and/or an increase in conversion rate [9]. Data on the timing of gametocytes appearing in the circulation can resolve this issue, but again, requires repeated sampling at specific time points. While there are important ethical and logistical considerations when studying natural infections of humans, monitoring infections, with measurements of conversion and in-host variables (e.g. anaemia and genetic diversity) would be extremely useful.

To address the problems outlined in points 1 and 2, ideally, conversion rates for rodent malaria parasites *in vivo* could be calculated in the same way as is now possible for *in vitro* cultures of *P. falciparum* (using GFP-tagged molecular markers of sexually committed schizonts and flow cytometry to sort fluorescent parasites [84])*.* However, despite the issues raised, measuring conversion rate remains a more desirable approach than simply analysing gametocyte density or prevalence, because changes in the density of gametocytes can be generated from cohorts that simply differ in asexual parasite number, but invest in the same relative number of gametocytes.

In multicellular organisms, reproductive effort decisions are based on multiple extrinsic and intrinsic cues, mortality risk and how these factors vary through an individual’s lifetime [25, 26, 29–31]. Evolutionary theory predicts organisms should invest less in reproduction as they age because deterioration in their physiological condition (referred to as ‘state’) means that more resources need to be allocated to maintenance to ensure continued survival [29–31]. However, when facing an irrecoverable decline in state, or other fatal circumstances, organisms should make a terminal investment to maximize short-term reproduction [29, 32, 33].

When translating this to malaria parasites each genotype within a mixed infection is the target of selection and should behave as a multicellular organism [34]. The density and/or proliferation rate of parasites is analogous to the ‘state’ of multicellular organisms. During infections, numerous factors, such as competition with unrelated genotypes, other species, drug treatment, immune responses, RBC resource availability and host nutritional status can all change dramatically and impact upon parasite proliferation in the host. Thus, in-host environmental factors that negatively affect proliferation can be considered as ‘stressors’ which impact on the ‘state’ of parasites.

## STRESS-INDUCED SEX?

Human (*Plasmodium falciparum*) and rodent (*Plasmodium chabaudi*) malaria parasites elevate gametocyte densities in response to high doses of antimalarial drugs [4–6, 35] and an increase in young RBCs (reticulocytes) [36, 37]. However, care must be taken when making comparisons as there are discrepancies between the approaches used to estimate conversion rates in different studies (Box 1). Increasing conversion has been interpreted as a strategy parasites adopt when they experience adverse conditions, enabling them to maximize transmission before the infection is cleared or the host dies [4, 8], a so-called ‘terminal investment’ [29]. While this makes intuitive sense in the case of drug treatment, it is not clear whether reticulocytes are, or indicate, adverse conditions.

In contrast, recent experiments (using *P. chabaudi* rodent malaria parasites *in vivo* [7, 38], and human *P. falciparum* parasites *in vitro* [39]) reveal that when exposed to competition with other genotypes in the host, RBC resource limitation, or low doses of anti-malarial drugs, parasites reduce conversion rates, adopting ‘reproductive restraint’ (Fig. 1). Evolutionary theory predicts that reproductive restraint during periods of mild stress improves the prospects for in-host survival, and therefore the opportunities for future transmission [40]. The experimental data also suggest that parasites respond to the presence of the extrinsic (environmental factors) as well as to their intrinsic effect (impact on state). Moreover, data from monitoring a cohort of infected patients collected in the same area from which the parasites used in Reece *et al*. [39] were isolated provide tentative (*in vivo*) support for the reproductive restraint of *P. falciparum* in response to drug pressure [41].

**Figure 2.**
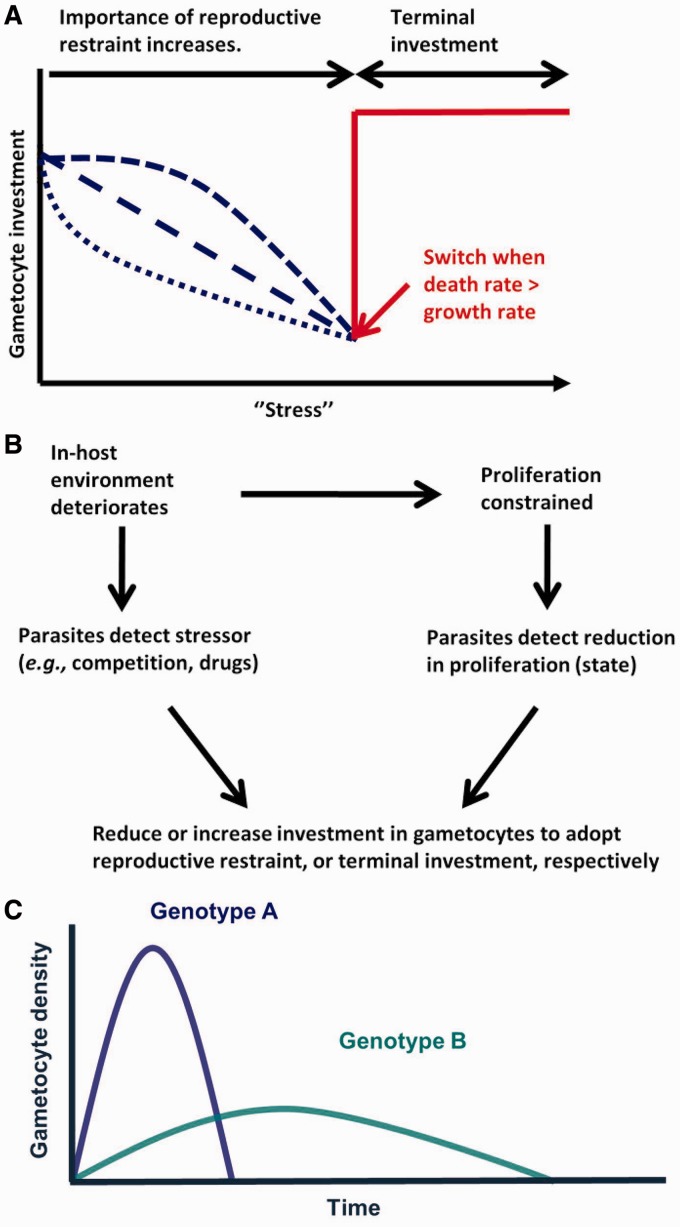
Predicted pattern for conversion. (**A)** Under low ‘stress’ (e.g. early in infections of naïve hosts) parasites can afford to invest in gametocytes, but if conditions deteriorate and proliferation is constrained (e.g. when parasites face stressors such as anaemia, competition or immune responses) parasites reduce conversion, employing reproductive restraint (blue dashed lines), to ensure in-host survival and the potential for future transmission. The form that reproductive restraint takes could follow any of the patterns illustrated with the dashed blue lines, depending on a number of factors (e.g. the cues parasites respond to, how accurately survival probability is determined, and the value of future versus current transmission). When parasites face circumstances likely to be fatal (e.g. when their death rate exceeds the potential for replication during radical drug treatment) or host death is imminent (e.g. due to severe anaemia), parasites should make a terminal investment by investing remaining resources into gametocytes (red solid line). A switch point and step function between reproductive restraint and terminal investment is predicted because investing all remaining resources is the best option in a situation likely to be fatal. Note: the *x*-axis does not simply translate to ‘time since infection’ because the severity of different stressors fluctuates during infections. (**B**) Data suggest that parasites can detect and respond directly to individual stressors and also to the effect they have on proliferation rate. Information from the cues parasites use must be fed into the molecular pathways that underpin commitment to effect a gametocyte investment decision. (**C**) The total production of gametocytes (the area under the curve) is equal for both genotypes [14]. However, genotype A invests heavily into transmission early in the infection and therefore achieves higher gametocyte densities over a shorter period of time, whereas B has a lower relative investment in gametocytes at each time point, but achieves a longer period for transmission. The optimal balance between these two extremes is predicted to depend on many factors including the frequency of vector blood meals, and the chances of the host clearing the infection or dying

The contrasting observations of increased and decreased conversion rates in response to environmental variation within the host can be reconciled by considering the severity of stress imposed on parasites by in-host factors. This is illustrated in Fig. 2A in which we propose that parasites adjust their conversion rate according to the impact of conditions on their proliferation (state) or via directly detecting the presence of stressors (Fig. 2B). In low stress conditions (e.g. infections of naïve hosts) parasites can afford to invest in gametocytes, and do so at a rate that maximizes transmission. When in-host conditions deteriorate due to the appearance of stressors (e.g. competition with other genotypes and species, immune responses, drug treatment), parasites are constrained to invest in survival, which they achieve by reducing the conversion rate (reproductive restraint) [23, 42]. By ensuring survival during periods of stress, parasites benefit from the fitness returns of future transmission (i.e. by reducing the rate of transmission in the short term, parasites gain a longer duration for transmission). When faced with attack from immune responses, investing more in replication may also have the added benefit of increasing opportunities for immune evasion via antigenic switching [43]. However, in very poor conditions, when parasites experience severe stress and their death rate exceeds the capacity for proliferation or host mortality is imminent, they should make a terminal investment to maximize short-term transmission by diverting resources to gametocyte production.

The pattern of conversion we predict in Fig. 2A is qualitatively similar to that predicted through a mathematical analysis by Koella and Antia [23]. Their analysis relied on strict assumptions: infections are lethal to the host above a threshold density and conversion rates are adjusted to limit asexual parasite densities to just below this threshold. This work raises the point that all else being equal, increasing investment in gametocytes should lead to decreasing virulence of an infection; a large body of theory predicts how virulence should depend on in-host factors (e.g. [15], reviewed in [44]). However, virulence is only one of the many selective forces acting on conversion rates. As only a small proportion of modern human malaria infections are fatal, we predict parasites more often need to respond to in-host factors that are able to clear infections than to imminent host death. The high prevalence of chronic malaria infections and the increasing appreciation of their contribution to the infectious reservoir [45–47], also suggests that a long duration of transmission matters and producing gametocytes ‘few but often’ results in the greatest lifetime fitness. Transmission success is also heavily dependent on vector availability. In areas where transmission is seasonal, parasites must survive in the host during the dry season. Indeed, parasites have evolved diverse strategies to facilitate long-term in-host survival, from immune evasion mechanisms (e.g. antigenic switching in *P. falciparum* [16]); to resisting competition (e.g. rodent malaria parasites prevent incoming, competing parasites from establishing an infection via the host iron regulatory hormone hepcidin [48]). In the majority of parasite species, the success of these strategies depends on maintaining asexual replication at a sufficiently high rate, which can be achieved through reproductive restraint.

## TESTING THE THEORY: COMPLICATIONS AND CHALLENGES

The model outlined above provides a foundation to explain variable conversion rates when considered in light of several key questions:

- Which cues do parasites use to make conversion rate decisions?
- What are the mechanisms that enable plasticity in conversion rate?
- How finely tuned are conversion rates to the in-host environment and state?
- Does adjusting conversion rates in the manner predicted maximize parasite fitness?

We consider answers to these questions in the following sections and outline the challenges required to evaluate these hypotheses in Box 2.

**Box 2: Challenges and future directions**

While our conceptual model is general, testing it requires examining specific circumstances. Here, we outline the main challenges and outstanding questions involved.

**Response to drugs:** Data for conversion rates—especially from experiments using drugs—are consistent with the basic prediction of parasites adopting reproductive restraint (*P. falciparum in vitro* [39]), or terminal investment (*P. chabaudi in vivo* [4, 6] and *P. falciparum in vitro* [5]), in response to different levels of stress. However, further work is required to explicitly test the effects of varying dose within the same experiment—both for rodent models and *in vitro* for *P. falciparum*. Furthermore, not all drugs appear to induce changes in conversion rate [6, 35]. This may be because drugs with different modes of action differentially affect the capacity of survivors to detect/respond to changes in state, or the capacity of dying parasites to provide signals.

**Response to competition:** In-host competition is a stressor with a negative effect on state because the densities of all genotypes (individually and when combined) is reduced in mixed infections compared with single infections. This is due to a mixture of competition for RBC and the action of immune repsonses that are not genotype specific. Competition within the host could occur via a single bite from a mosquito infected with multiple genotypes (to a naive host). Alternatively, competition can be established when a mosquito infected with one genotype bites an individual already infected with a different genotype. The latter example of sequential infection would be less stressfull for the resident genotype than the newcomer, even if it the resident genotype is competitively inferior to the incoming genotype [85]. This is because the incoming genotype will enter a RBC resource depleted environment with cross-reactive immune responses already in place [86]. *In vivo* studies of simultaneous in-host competition using *P. chabaudi* reveal reproductive restraint across several genotypes [7, 87], but there are no reports of increased conversion in response to competition. Adopting reproductive restraint in response to competition might be the only strategy required because in-host competition is never stressful enough to merit terminal investment. Alternatively, this may be an artefact of experimental design in which mixed infections do not result in competitive exclusion, even for the weakest genotypes [7, 88, 89]. Experiments using genotypes that vary in competitive ability, inoculated at different starting doses and times during infections are needed to test whether in-host competition can induce terminal investment. At the host population level, the consequences of different investment strategies would be much harder to test experimentally, but theory demonstrates that there will be feedback from the within- to between-host levels, and vice versa (e.g. [90]). For instance, if mixed infections really do promote reproductive restraint, then this should result in less transmission and, consequently, fewer mixed infections. Some of the variations observed in conversion rates may be a consequence of this sort of dynamic feedback.

**Response to reticulocytes:** Conversion has been observed to both increase and decrease in response to reticulocytes. For some species (e.g. *P. berghei* and *P. vivax*) that preferentially invade reticulocytes, an increase in conversion upon exposure to reticulocytes is consistent with parasites making use of available resources. However, species able to infect a wide range of RBC ages, such as *P. falciparum* and *P. chabaudi,* also increase conversion in response to reticulocytes [36, 37]. This may be because reticulocytes are also exploitable resources. However, the lifespan of gametocytes in *P. falciparum* is at least five times that of asexual stages, so the longer expected lifespan of reticulocytes may provide a better resource to support the development of gametocytes than mature RBCs. Alternatively, for all species, increased reticulocytaemia could indicate severe anaemia leading to imminent host death, and thus, terminal investment is the best strategy. For example, the poultry malaria parasite *P. gallinaceum* appears to be able to determine whether the host will survive or die from severe anaemia because it produces different sex ratios in these different circumstances [55]. However, an influx of reticulocytes could also indicate the opposite—that the host is generating an appropriate erythropoietic response and will recover from severe anaemia. In this case, reproductive restraint maximizes the potential for the parasites to survive.

When in-host survival does not rely on asexual parasite replication: Parasite species producing dormant stages that persist in the liver (hypnozoites) and dendritic cells, such as the human malaria parasites *P. vivax* and *P. ovale* [91, 92], may not adopt reproductive restraint in response to stress because survival in the host does not depend on blood stage replication. Terminal investment due to imminent clearance will also be unnecessary but may be required to cope with host death. To our knowledge there are no data on the conversion rates of *P. vivax* experiencing different in-host conditions. However, during natural *P. vivax* infections, higher gametocyte densities are correlated with a mixture of seemingly favourable and unfavourable conditions, including younger (immunologically naive) patients, those with higher parasite densities, lower haemoglobin levels, lower platelet counts and an absence of fever (reviewed in [12]). *Plasmodium vivax* gametocyte densities are also generally much higher compared with those recorded for *P. falciparum*, but each gametocyte circulates for a shorter time; a maximum of 3 days (reviewed in [12]). These observations suggest that *P. vivax* may have a non-plastic strategy of a relatively high conversion during the short-lived erythrocytic stage of their infections.

### Cues for conversion decisions

The extent to which parasites respond directly to extrinsic stressors or simply the overall effect those stressors have on state is not known. Experimental data suggest parasites can respond both to state and environmental factors. For example, experiments exposing *P. falciparum* to low doses of different anti-malarial drugs in culture have included both drug sensitive and resistant genotypes but only sensitive genotypes respond. This suggests that parasites do not directly detect each drug, but instead, respond to the negative effect they have on state [39]. Responding to state seems the more efficient strategy: it avoids the need to integrate information about multiple factors, potentially giving opposing information, to mount an appropriate response. For example, the level of anaemia induced by *P. falciparum* infections varies depending on the type of antimalarial drug administered to patients and whether the parasites are cleared [49]. Because anaemia triggers the formation of reticulocytes, the reproductive strategy employed in response to the presence of drugs may be complicated by the simultaneous change in RBC age structure. Parasites could be responding directly to the drugs, the resulting changes in RBCs, both, or the overall effect that both factors have on the ‘state’ of the infection [36, 37].

Whether the best measure of state is parasite density *per se* or proliferation (i.e. rate of change in density) is unclear. Data from several *P. chabaudi* genotypes [7, 50] and subsequent modelling [51] suggests that parasites alter their conversion rate according to their density in mixed genotype infections. Density could be determined by quorum sensing [52], markers of RBC lysis from burst parasitized cells [53], immune factors, or metabolic measures such as energy balance or reducing power (e.g. the expression of genes associated with starvation are associated with increased conversion in *P. falciparum* [54]). However, detecting the density of a parasite cohort does not necessarily reveal a change in state (i.e. is parasite density increasing or decreasing?).

Measuring proliferation requires that parasites integrate information on density over consecutive cell cycle cohorts. This information may be more accurate for parasite species with synchronous progression through cell cycles than for species with asynchronous cycles. In this case, if proliferation rate information is unreliable, parasites could respond to individual environmental stress factors; either directly or indirectly, by detecting a co-varying factor. For example, parasites may use the onset of anaemia as a signal for the imminent arrival of antibodies and the development of immune responses [37, 55, 56]. Using proxies in this way may also enable parasites to predict future changes in state and respond preemptively [57]. Alternatively, parasites could measure their death rate; although mechanisms for this are more difficult to envision, they could include monitoring the concentration of immune effectors or the release of SOS signals by dying parasites similar to bacteria and *Chlamydomonas* [58, 59].

### Mechanisms underpinning conversion

The mechanisms regulating the switch to gametocyte production remain elusive. Advances in genomics, transcriptomics, proteomics and functional gene targeting studies have identified several markers of early gametocyte development in human and rodent malaria parasites (reviewed in [8, 10, 60–63]). These studies provide further evidence that commitment occurs at or prior to the schizont stage preceding the release of sexually committed merozoites (as has been previously suggested for *P. falciparum* [64, 65]). Studies using GFP reporters with known gametocyte specific promoters also support this developmental pattern (reviewed in [10, 13, 60, 61]). Recently, the gene *P. falciparum* gametocyte development 1: Pfgdv1 (PFI1710w) has been identified as a regulator of gametocyte production (and is associated with an increased expression of genes involved in early gametocytogenesis (Pfge genes) [66]), and work from our group has identified an ApiAP2 DNA binding protein [67] that is required for gametocyte commitment (Kafsack and Llinás, unpublished data).

While identifying molecular markers for commitment is useful for quantifying conversion decisions, the evolution of plasticity in conversion rates is shaped by the nature of the pathways involved in: detecting cues, processing the information, producing a conversion rate phenotype and the maturation of gametocytes. The critical regulators underlying gametocyte conversion may act within a complex network of interactions between different modules involved in information assimilation and integration to produce a conversion rate phenotype. This level of complexity is very challenging to unravel and made more difficult because gene function and changes in expression must be assessed in the context of variation in both the environment and genetic background of the parasites. Furthermore, it is possible that the environmental sensing mechanisms underlying conversion decisions may also feed information into other plastic life history decisions such as sex ratio, cell cycle arrest and var gene switching (which is responsible for antigenic variation to evade host immune responses), as these traits are sensitive to similar environmental perturbations (reviewed in [24]). As these traits are likely to be linked by genetic correlations (e.g. epistasis/pleiotropy: different traits are shaped by the same genes), understanding the nature of these interactions is central to explaining plasticity in these traits.

### Parameterizing patterns of conversion

The shape and switch point(s) of the reaction norm (how a trait varies across an environmental gradient) reveal how fine-tuned parasite responses are to environmental variation, including novel stressors. The extent of genetic variation for reaction norms is a determinant of the potential for evolution. Reaction norms are influenced by many interacting factors. This includes the reliability of cues, costs of maintaining detection and response mechanisms, and how much multiple sources of information affect the risk of making the wrong decision [68–70]. Differences in reaction norms across species, that have different cell-cycle durations, gametocyte development times or RBC age preferences, may reflect how differences in costs and constraints on plasticity shape parasite strategies. As many different factors can independently and simultaneously affect in-host conditions and parasite state, examining the patterns of conversion rates resulting from varying factors individually is useful, but providing cues in different combinations is required to reveal the full picture.

The reaction norm for conversion is predicted to follow a non-linear pattern, with any of the patterns illustrated and at least 1 switch point (reproductive restraint to terminal investment; Fig. 2A) [23, 42]. This switch should occur when the death rate exceeds the proliferation rate. We expect this point will be influenced by species-specific variation in cell-cycle duration and gametocyte development time, and by how quickly the environment and/or state changes. For example, the cell-cycle duration and gametocyte development time of rodent malarias are much shorter than that of *P. falciparum.* While the cell cycle for rodent malaria parasites is 24 hours, and gametocytes reach maturity and are infectious to mosquitoes after 24–48 hours, the cell cycle of the human malaria parasite *P. falciparum* is 48 hours and gametocytes require 10–14 days to reach maturity [10, 11]. Therefore, if *P. falciparum* makes a terminal investment in advance of host death the host is required to survive at least 10–14 days until the investment can pay off (five further asexual cycles), but only 48 hours are required for rodent parasites to produce transmissible gametocytes. As such, *P. falciparum* may ‘play it safe’ and adopt a more conservative strategy by making a terminal investment in response to lower levels of stress than rodent parasites, whose gametocytes reach maturity within 48 hours (two asexual cycles). If a fast drop in numbers were normally a reliable indicator of a terminal situation, this would explain why increased conversion is observed when parasites are exposed to high, but subcurative, drug doses [39, 41]. Also, if the longer cell-cycle duration of *P. falciparum* compared to rodent malarias makes *P. falciparum* more vulnerable to being cleared by the host, reproductive restraint will be induced at lower stress than for rodent parasites.

As shown in Fig. 2C, the characteristics of populations can also influence the shape of reaction norms. For example, a ‘live fast, die young’ strategy in which parasites readily switch to terminal investment may bring greater pay offs in an epidemic setting—where there are plenty of naïve hosts to be transmitted to—than in an endemic setting where parasites will be transmitted to hosts containing competitors and with active immune responses [71]. This is because genotypes with a high conversion rate risk being unable to establish infections in new hosts, due to being outcompeted by resident genotypes [15, 40]. Furthermore, Parasites in hypoendemic areas experience lower levels of in-host competition than those from regions with high genetic diversity (hyperendemic) and so may be less responsive to novel stressors such as competition and its effect on state.

### Linking variable conversion rates to fitness

A key prediction to test is whether plasticity in conversion rate is adaptive [72]. The extent to which reproductive restraint provides an in-host survival advantage under stress is yet to be determined (e.g. how much does reproductive restraint ameliorate the suppression of a genotype in a mixed infection?). At the between-host level, how different reproductive strategies map to the rate and duration of transmission is hard to assess from data (e.g. gametocyte prevalence) available on natural infections. Therefore, whether (under some conditions) prolonging the duration for transmission enhances fitness, and whether terminal investment benefits parasites in lethal situations through an increase in short-term transmission, remain unknown.

Testing the fitness consequences of variation in traits is notoriously difficult, but identifying the host and parasite factors that elicit a change in conversion rate and the reaction norms generated by different levels of stress will provide the required foundations. For example, by providing a cue that elicits reproductive restraint in different circumstances (e.g. cues for competition provided in single infections) parasites can be induced (‘tricked’) into making inappropriate responses for their circumstances. The consequences for in-host survival and transmission for parasites responding to fake cues could then be quantified, and compared to the performance of parasites exposed to cues that accurately reflect their circumstances [73]. This framework also opens up the possibility of developing interventions that co-opt plasticity in conversion rates, by manipulating parasites into making suboptimal decisions for their fitness.

The maintenance of mechanisms required to detect and respond to environmental change requires resources that could be otherwise allocated to different functions [74]. Evolutionary theory predicts that if these costs are sufficiently high then plasticity is selected against and lost if organisms no longer experience variable environments, but evidence for costs of plasticity is scarce [75]. Because gametocytes are costly, selection for in-host replication during long-term culture of *P. falciparum* and serial passage of *P. berghei* result in the loss of gametocyte production [8, 76]. However, whether plasticity is actually lost is unclear because gametocyte production is sometimes recoverable [77].

## CONCLUSIONS

That in-host ecology shapes the dynamics of infections [78, 79] and patterns of transmission is well known [12, 80–82]. Despite this, why the density of circulating gametocytes in malaria is generally low [9, 40] has eluded explanation. We provide an evolutionary theory-based model, which predicts that parasites can rarely afford to invest in more because their life history spreads reproduction across multiple attempts over a relatively long time period.

Given renewed interest in transmission blocking interventions, understanding parasite strategies for gametocyte investment is central to making such measures as resilient to parasite counter evolution as possible [12, 15]. For example, inducing all parasites to commit to gametocytes (ideally of the same sex) would reduce the virulence of the infection and could also produce an effective transmission-blocking immune response that acts against future infections. For example, this could be useful for travellers returning to non-malarious countries. Inducing commitment *in vitro* could also generate material to inform the development of other transmission-blocking interventions such as vaccines and drugs with gametocytocidal action.

Finally, it is often not appreciated that plasticity in parasite life history traits can also shape evolutionary responses to environmental change. For example, if plasticity in conversion rate acts as a buffering mechanism to minimize the impact of drug treatment, this may weaken selection for other forms of resistance. This may be favourable from the perspective of maximizing the timespan of efficacy of antimalarial drugs. However, such infections will likely be harder to treat than if malaria parasites exhibited a higher, fixed, conversion rate.
